# Supplementary material for: Content-rich biological network constructed by mining PubMed abstracts
Source: BMC Bioinformatics. 2004 Oct 8;5:147. doi: 10.1186/1471-2105-5-147 (PMC528731; doi:10.1186/1471-2105-5-147)
Supplement: Additional File 2 — The original results of the above study (non-essential files are deleted to keep the file size under the limit set by BMC bioinformatics). [file 1471-2105-5-147-S2.bz2 › chilibotAdditionalFile2/dip50/217ID7744001E725/html/IL6ST_IL6.html]

 


 **IL6ST** and **IL6** 
  
Found 848 abstracts in PubMed, retrieved 50.  
 

 What does Google say? 
 PDF only 
| .edu only 

---

**Interactive relationship** (e.g. stimulation, inhibition, etc)

**Inhibitory relationship**- Osteoclastogenesis induced by IL 6  [ **IL6** ]  and IL 11 was inhibited by the addition of an anti gp130  [ **IL6ST** ]  antibody but not by osteoprotegerin.  Ref: 12584029 Bone, 2003
- In this respect murine fibroblasts lacking exon 3 of SHP2 are not sensitive to TNF alpha, indicating that functional SHP2 and its recruitment to gp130  [ **IL6ST** ]  are key events in inhibition of IL 6  [ **IL6** ]  dependent STAT activation by TNF alpha.  Ref: 12817006 J Immunol, 2003
- The chimera induced increase of oligodendrocyte arborization was mimicked, although with lower efficacy, by ciliary neurotrophic factor but not by IL 6  [ **IL6** ]  and was reduced in the presence of a gp130  [ **IL6ST** ]  soluble peptide which is able to inhibit the gp130  [ **IL6ST** ]  mediated signals of the IL 6  [ **IL6** ]  sIL 6R complex.  Ref: 12504593 Mol Cell Neurosci, 2002
- We previously demonstrated that interleukin 6  [ **IL6** ]  related cytokine, leukemia inhibitory factor LIF, induced cardiac hypertrophy through gp130  [ **IL6ST** ] .  Ref: 12855672 Circ Res< MedlineTA>Circ Res, 2002
- gp130  [ **IL6ST** ]  is a signal transducing receptor component shared by all the IL 6  [ **IL6** ]  family cytokines, whose gene ablation in mouse results in the significant reduction in the fetal liver hematopoiesis.  Ref: 12691915 Exp Hematol, 2003
- Leukemia inhibitory factor LIF, oncostatin M OSM, interleukin IL 6  [ **IL6** ] , and IL 11 regulate hemopoiesis and LIF, OSM, and IL 6  [ **IL6** ]  also inhibit the proliferation of myeloid leukemic cell lines through the signal transducing subunit gp130  [ **IL6ST** ] .  Ref: 12543079 Cytokine, 2002
- Sant 7 is a mutated form of IL 6  [ **IL6** ]  that can bind to the IL 6R, but inhibits its ability to interact with the gp130  [ **IL6ST** ]  signal transducing protein.  Ref: 12592380 Br J Cancer, 2003
**Neutral relationship**- Synthetic peptides mimicking the interleukin 6  [ **IL6** ]  gp130  [ **IL6ST** ]  interaction a two helix bundle system.  Ref: 12630694 J Pept Sci, 2003
- AIMS Interleukin 6  [ **IL6** ]  IL 6  [ **IL6** ]  contributes via its signal transducer gp130  [ **IL6ST** ]  to the acute phase response APR in hepatocytes.  Ref: 12891556 Gastroenterology, 2003
- Ciliary neurotrophic factor CNTF is a gp130  [ **IL6ST** ]  neuroregulatory cytokine belonging to the interleukin 6  [ **IL6** ]  type cytokine superfamily.  Ref: 12890930 Gynecol Obstet InvestGynecol Obstet Invest, 2003
- Cytokines of the interleukin 6  [ **IL6** ]  family utilize the shared cytokine receptor gp130  [ **IL6ST** ]  in the formation of active signalling complexes.  Ref: 12593670 Biochem J, 2003
- Thus, constitutive activation of STAT3 in HNSCC may use an autocrine paracrine activating loop mediated by  **IL6**  and other cytokines acting through the gp130  [ **IL6ST** ]  receptor family, which may confer both proliferative and survival potential in this malignancy.  Ref: 12782602 Cancer Res, 2003
- IL 11 belongs to the gp130  [ **IL6ST** ]  cytokine family, which includes also IL 6  [ **IL6** ] .  Ref: 12697042 J Endocrinol, 2003
- The IL 6  [ **IL6** ]  receptor is composed of two different subunits, an alpha subunit IL 6Ralpha that binds IL 6  [ **IL6** ]  with low affinity and a beta subunit gp130  [ **IL6ST** ]  that binds the IL 6  [ **IL6** ] .IL 6Ralpha complex with high affinity and as a result triggers intracellular signaling.  Ref: 12646580 J Biol Chem, 2003
- In its soluble form, gp130  [ **IL6ST** ]  is a natural antagonist that neutralizes IL 6  [ **IL6** ] .soluble IL 6Ralpha complexes.  Ref: 12646580 J Biol Chem, 2003
- One good illustration is derived from the in vitro observations that IL 6  [ **IL6** ]  promotes the growth arrest and differentiation of M1 cells through gp130  [ **IL6ST** ]  mediated STAT3 activation.  Ref: 12687404 Rev Physiol Biochem Pharmacol< MedlineTA>Rev Physiol Biochem Pharmacol, 2003
- Previous studies showed that activation of the gp130  [ **IL6ST** ]  receptor system, using as ligand interleukin 6  [ **IL6** ]  fused to its soluble receptor IL6RIL6, causes induction of myelin genes such as myelin basic protein MBP and myelin protein zero Po in embryonic dorsal root ganglia Schwann cells.  Ref: 12517919 J Biol Chem< MedlineTA>J Biol Chem, 2003
- Previous studies showed that activation of the gp130  [ **IL6ST** ]  receptor system, using as ligand interleukin 6  [ **IL6** ]  fused to its soluble receptor IL6RIL6, causes induction of myelin genes such as myelin basic protein MBP and myelin protein zero Po in embryonic dorsal root ganglia Schwann cells.  Ref: 12643284 J Biol Chem, 2003
- BACKGROUND Experimental studies indicate that interleukin 6  [ **IL6** ]  IL 6  [ **IL6** ]  related cytokines, signaling via the shared receptor gp130  [ **IL6ST** ] , Janus kinases JAKs, and signal transducers and activators of transcription STATs, provide a critical cardiomyocyte survival pathway in vivo.  Ref: 12591746 Circulation, 2003
- The second nonligand binding chain of IL 6  [ **IL6** ]  receptor IL 6R, the membrane glycoprotein with 130 kD mol wt gp130  [ **IL6ST** ] , is responsible for the signal transduction of IL 6  [ **IL6** ]  biological activity.  Ref: 12513832 Zhongguo Shi Yan Xue Ye Xue Za Zhi, 2002
- LIF acts on responding cells by binding to a heterodimeric membrane receptor composed of a low affinity LIF specific receptor and the gp130  [ **IL6ST** ]  receptor chain also used as the receptor for interleukin 6  [ **IL6** ] , oncostatin M, cardiotrophin 1, and ciliary neurotrophic factor.  Ref: 12529546 Stem Cells, 2003
- High circulating levels of biologically inactive IL 6  [ **IL6** ]  SIL 6 receptor complexes in systemic juvenile idiopathic arthritis evidence for serum factors interfering with the binding to gp130  [ **IL6ST** ] .  Ref: 12562400 Clin Exp Immunol, 2003
- In conclusion, our experiments indicate an involvement of IL 6  [ **IL6** ]  in the pathogenesis of liver diseases and suggest a protective role of IL 6  [ **IL6** ]  gp130  [ **IL6ST** ]  dependent pathways in nonparenchymal liver cells during fibrosis progression in chronic liver diseases.  Ref: 12830005 Hepatology< MedlineTA>Hepatology, 2003
- Interleukin 6  [ **IL6** ]  IL 6  [ **IL6** ]  is an immunoregulatory cytokine that activates a cell surface signaling assembly composed of IL 6  [ **IL6** ] , the IL 6  [ **IL6** ]  alpha receptor IL 6Ralpha, and the shared signaling receptor gp130  [ **IL6ST** ] .  Ref: 12829785 Science, 2003
- In a target cell, IL 6  [ **IL6** ]  can simultaneously generate functionally distinct or sometimes contradictory signals through its receptor complex, IL 6Ralpha and gp130  [ **IL6ST** ] .  Ref: 12687404 Rev Physiol Biochem Pharmacol< MedlineTA>Rev Physiol Biochem Pharmacol, 2003
- The effects of IL 6  [ **IL6** ]  are mediated through a specific receptor complex made up of a ligand binding glycoprotein gp80 or IL 6R and a signal transducing glycoprotein gp130  [ **IL6ST** ] .  Ref: 12664314 Eur Arch Psychiatry Clin Neurosci, 2003
- IL 6  [ **IL6** ]  acts by binding to a low affinity membrane spanning receptor IL 6R, which must associate with a high affinity receptor gp130  [ **IL6ST** ]  for signal transduction to occur.  Ref: 12592380 Br J Cancer, 2003
- The subsequent recruitment of the common signal transducer gp130  [ **IL6ST** ]  and activation of the JAK STAT and SHP 2 Ras mitogen activated protein kinase MAPK pathways are responsible for the majority of cellular responses elicited by IL 6  [ **IL6** ]  cytokines.  Ref: 12764151 J Biol Chem< MedlineTA>J Biol Chem, 2003
- Using an assay for the measurement of the amount of circulating IL 6  [ **IL6** ]  complexed with the sIL 6R and available for binding to gp130  [ **IL6ST** ]  gp130  [ **IL6ST** ]  binding activity, we show that the IL 6  [ **IL6** ]  gp130  [ **IL6ST** ]  binding activity is similar to that detected by the bioassays and again significantly lower than that estimated to be present in the IL 6  [ **IL6** ]  sIL 6R complex.  Ref: 12562400 Clin Exp Immunol, 2003
- IL 6  [ **IL6** ]  type cytokines exert their action via the signal transducers gp130  [ **IL6ST** ] , LIF receptor and OSM receptor leading to the activation of the JAK STAT and MAPK cascades.This review focuses on recent progress in the understanding of the molecular mechanisms of IL 6  [ **IL6** ]  type cytokine signal transduction.  Ref: 12773095 Biochem J< MedlineTA>Biochem J, 2003
- The mRNAs of both receptors of IL 6  [ **IL6** ] , the soluble IL6R and gp130  [ **IL6ST** ] , were increased subsequently to IL 6  [ **IL6** ]  mRNA.  Ref: 12733076 Pflugers Arch< MedlineTA>Pflugers Arch, 2003
- Addition of recombinant human IL 6  [ **IL6** ]  rhIL 6 to sera of patients or controls results in a markedly lower increase in the gp130  [ **IL6ST** ]  binding activity in patients than in controls.  Ref: 12562400 Clin Exp Immunol, 2003

**Non-interactive relationship** (e.g. studied together, co-existance, homology, etc.)

- In the non lineage specific gp130  [ **IL6ST** ]  cytokine family, IL 6  [ **IL6** ]  appears to predominate for the induction of megakaryopoiesis.  Ref: 12634936 Rheumatol Int, 2003
- Here we show that phosphorylation of STAT3 is prolonged in mouse Socs3 deficient macrophages after stimulation with interleukin 6  [ **IL6** ]  IL 6  [ **IL6** ]  but not IL 10, indicating that SOCS3 specifically affects signaling mediated by IL 6  [ **IL6** ]  and gp130  [ **IL6ST** ] .  Ref: 12754506 Nat Immunol< MedlineTA>Nat Immunol, 2003
- To define the IL 6  [ **IL6** ]  dependent intracellular pathways more specifically, we generated a hepatocyte specific gp130  [ **IL6ST** ]  knockout mouse.  Ref: 12891556 Gastroenterology, 2003
- Interleukin 6  [ **IL6** ]  IL 6  [ **IL6** ]  activates cells by binding to the membrane bound IL 6  [ **IL6** ]  receptor IL 6R and subsequent formation of a gp130  [ **IL6ST** ]  homodimer.  Ref: 12832423 J Biol Chem< MedlineTA>J Biol Chem, 2003
- In addition to CNTF, the mRNAs of leukemia inhibitory factor, interleukin 6  [ **IL6** ] , ciliary neurotrophic factor receptor component alpha and gp130  [ **IL6ST** ]  were detected in the Schwann cells.  Ref: 12618289 Neurosci Lett, 2003
- LPS stimulation in gp130  [ **IL6ST** ]  deleted and also  **IL6**  animals after PH leads to a significant reduction in survival and DNA synthesis, which was associated with decreased Bcl xL expression and higher apoptosis in the liver.  Ref: 12509437 J Biol Chem, 2003
- Furthermore, activation of p38 mitogen activated protein kinase is shown to be essential for the inhibitory effect of TNF alpha on IL 6  [ **IL6** ]  signaling and TNF alpha dependent recruitment of SHP2 to gp130  [ **IL6ST** ] .  Ref: 12817006 J Immunol, 2003
- Epidermal growth factor receptor independent constitutive activation of STAT3 in head and neck squamous cell carcinoma is mediated by the autocrine paracrine stimulation of the interleukin 6  [ **IL6** ]  gp130  [ **IL6ST** ]  cytokine system.  Ref: 12782602 Cancer Res, 2003
- ELISA was used to determine the serum levels of soluble interleukin 2 receptors, interleukin 1, interleukin 6  [ **IL6** ] , and 2 soluble interleukin 6  [ **IL6** ]  receptors sIL 6R and soluble gp130  [ **IL6ST** ]  in 18 patients with cluster headache 6 women and 12 men during the cluster period and in 17 healthy controls who were headache free 3 women and 14 men.  Ref: 12864761 Headache, 2003
- In vivo activation of the interleukin 6  [ **IL6** ]  receptor gp130  [ **IL6ST** ]  signaling pathway in pituitary corticotropes of lipopolysaccharide treated rats.  Ref: 12624539 Neuroendocrinology, 2003
- Thus, the data reported herein represent a promising indication on how to develop new peptides able to interfere with formation of the interleukin 6  [ **IL6** ]  gp130  [ **IL6ST** ]  complex.  Ref: 12630694 J Pept Sci, 2003
- Since recruitment of the tyrosine phosphatase Src homology protein tyrosine phosphatase 2 SHP2 to the signal transducing receptor subunit gp130  [ **IL6ST** ]  attenuates IL 6  [ **IL6** ]  mediated STAT activation, we were interested in whether TNF alpha also induces the association of SHP2 to the gp130  [ **IL6ST** ]  receptor subunit.  Ref: 12817006 J Immunol, 2003
- Cells that express gp130  [ **IL6ST** ] , but not the IL 6R, can be activated by IL 6  [ **IL6** ]  and the soluble IL 6R sIL 6R which is generated by shedding from the cell surface or by alternative splicing.  Ref: 12832423 J Biol Chem< MedlineTA>J Biol Chem, 2003
- Hexameric structure and assembly of the interleukin 6  [ **IL6** ]  IL 6  [ **IL6** ]  alpha receptor gp130  [ **IL6ST** ]  complex.  Ref: 12829785 Science, 2003
- after IL 6  [ **IL6** ]  and OSM stimulation, gp130  [ **IL6ST** ]  dependent pathways STAT3, APR gene expression were completely blocked in the liver of these animals.  Ref: 12891556 Gastroenterology, 2003
- To determine whether the pathophysiological processes after transient forebrain ischemia are mediated via a signal pathway involving gp130  [ **IL6ST** ]  a signal transducer for the interleukin 6  [ **IL6** ]  family, we analyzed changes in the expression of gp130  [ **IL6ST** ]  and its downstream transcription factor, signal transducer and activator of transcription factor 3 STAT3, in the rat hippocampus of a four vessel occlusive ischemia model.  Ref: 12528179 Glia, 2003
- A Fusion Protein of the gp130  [ **IL6ST** ]  and Interleukin 6Ralpha Ligand binding Domains Acts as a Potent Interleukin 6  [ **IL6** ]  Inhibitor.  Ref: 12646580 J Biol Chem, 2003
- Dual in situ hybridization revealed that most corticotropes expressed IL 6  [ **IL6** ]  receptor and gp130  [ **IL6ST** ]  mRNAs,.  Ref: 12624539 Neuroendocrinology, 2003
- This inhibitory activity does not appear to be due to soluble gp130  [ **IL6ST** ] , C reactive protein or autoantibodies to IL 6  [ **IL6** ] .  Ref: 12562400 Clin Exp Immunol, 2003
- These results show that sera from patients with s JIA contain a factor, or factors, that inhibit s the binding of the IL 6  [ **IL6** ]  sIL 6R complex to gp130  [ **IL6ST** ] .  Ref: 12562400 Clin Exp Immunol, 2003
- The objective of our study was to mimic in a typical reductionist approach the molecular interactions observed at the interface between the gp130  [ **IL6ST** ]  receptor and interleukin 6  [ **IL6** ]  during formation of their complex.  Ref: 12630694 J Pept Sci, 2003
- Here we studied the expression of cytokines IL 6  [ **IL6** ] , LIF, IL 1beta and TNFalpha and cytokine receptors IL 6R, LIFR and Gp130  [ **IL6ST** ]  in the rat brain after kainic acid induced status epilepticus using Northern blot analysis and in situ hybridization histochemistry.  Ref: 12591161 Brain Res Mol Brain Res, 2003
- However, in situations where a signaling competent receptor is bound at IL 6  [ **IL6** ]  site I, ligand binding to site III is an absolute requirement for participation of the receptor in a signaling heterodimer with gp130  [ **IL6ST** ]  ; i.e., a functional receptor complex of IL 6  [ **IL6** ]  type cytokines cannot be assembled solely via site I and II as in the growth hormone receptor complex.  Ref: 12731865 Biochemistry, 2003
- TNF alpha Induces Tyrosine Phosphorylation and Recruitment of the Src Homology Protein Tyrosine Phosphatase 2 to the gp130  [ **IL6ST** ]  Signal Transducing Subunit of the IL 6  [ **IL6** ]  Receptor Complex.  Ref: 12817006 J Immunol, 2003
- Thus, IL 6  [ **IL6** ]  gp130  [ **IL6ST** ]  signaling will become a more attractive therapeutic target for human inflammatory diseases when a better understanding of IL 6  [ **IL6** ]  signaling, including the identification of the conductor for gp130  [ **IL6ST** ]  signal transduction, is achieved.  Ref: 12687404 Rev Physiol Biochem Pharmacol< MedlineTA>Rev Physiol Biochem Pharmacol, 2003
- In contrast to the IL 6  [ **IL6** ]  signal transducer gp130  [ **IL6ST** ] , which has been previously shown to recruit SOCS3 to one of its phosphotyrosine residues Y759, peptide precipitation experiments suggest that SOCS3 does not interact with phosphorylated tyrosine motifs of the IL 10R.  Ref: 12626585 J Immunol, 2003
- Interleukin 6  [ **IL6** ]  gp130  [ **IL6ST** ]  dependent pathways are protective during chronic liver diseases.  Ref: 12830005 Hepatology< MedlineTA>Hepatology, 2003
- Interleukin 6  [ **IL6** ]  glycoprotein 130  [ **IL6ST** ]  dependent pathways are protective during liver regeneration.  Ref: 12509437 J Biol Chem, 2003
- In this context the cytoplasmic SHP2 SOCS3 recruitment site of gp130  [ **IL6ST** ]  tyrosine 759 is shown to be important for the inhibitory effects of TNF alpha, since mutation of this residue completely restores IL 6  [ **IL6** ]  stimulated activation of STAT3 and, consequently, of a STAT3 dependent promoter.  Ref: 12817006 J Immunol, 2003
- Fibroblasts Isolated from Normal Lungs and Those with Idiopathic Pulmonary Fibrosis Differ in Interleukin 6  [ **IL6** ]  gp130  [ **IL6ST** ]  Mediated Cell Signaling and Proliferation.  Ref: 12819039 Am J Pathol, 2003
- Using the recently engineered designer cytokine IC7, which induces an active heterodimer of the LIFR and gp130  [ **IL6ST** ]  after binding to the IL 6R, and several receptor chimeras of the LIFR and the interleukin 6  [ **IL6** ]  receptor IL 6R carrying the CBM of the IL 6R in place of the COOH terminal LIFR CBM, we could assign individual receptor subdomains to individual binding sites of the ligand.  Ref: 12731865 Biochemistry, 2003
- In macrophages lacking the Socs3 gene or carrying a mutation of the SOCS3 binding site in gp130  [ **IL6ST** ] , the lipopolysaccharide induced production of tumor necrosis factor TNF and IL 12 is suppressed by both IL 10 and IL 6  [ **IL6** ] .  Ref: 12754507 Nat Immunol< MedlineTA>Nat Immunol, 2003
- To prove the relevance of these clinical findings, we applied the tetrachlorcarbonide CCl 4 model to conditional knockout animals Cre loxP system for gp130  [ **IL6ST** ] , the common signal transducer of IL 6  [ **IL6** ]  family cytokines.  Ref: 12830005 Hepatology< MedlineTA>Hepatology, 2003
- To define better the role of  **IL6**  and its family members for liver regeneration after PH, we used conditional knockout mice for glycoprotein 130  [ **IL6ST** ]  gp130  [ **IL6ST** ] , the common signal transducer of all  **IL6**  family members.  Ref: 12509437 J Biol Chem, 2003
- BACKGROUNDLeukemia inhibitor factor LIF, a member of the interleukin 6  [ **IL6** ]  cytokine family, has recently been shown to inhibit HIV 1 replication both in vivo and in vitro.OBJECTIVELIF and its corresponding receptors gp130  [ **IL6ST** ]  and LIF receptor alpha LIFR alpha were studied in lymphoid tissue LT to reveal potential systemic immunoregulatory effects during the course of HIV 1 infection.METHODSLIF, gp130  [ **IL6ST** ] , LIFR alpha and HIV 1 replicating cells were identified at the single cell level by immunohistochemistry and quantified by computerized in situ imaging in tonsil and lymph nodes biopsies LT from patients with primary HIV 1 infection PHI, chronic HIV 1 infection cHI, long term non progressors LTNP and HIV 1 seronegative controls.RESULTSLIF and its receptors, gp130  [ **IL6ST** ]  and LIFR alpha were significantly P &lt.  Ref: 12799551 AIDS< MedlineTA>AIDS, 2003
- Although IL 6  [ **IL6** ]  gp130  [ **IL6ST** ]  signaling is a promising target for drug discovery for many human diseases, the interdependence of each signaling pathway may be an obstacle to the development of a nonpeptide orally active small molecule to inhibit one of these IL 6  [ **IL6** ]  signaling cascades, because it would disturb the signal orchestration.  Ref: 12687404 Rev Physiol Biochem Pharmacol< MedlineTA>Rev Physiol Biochem Pharmacol, 2003
- A peptide system obtained by reproducing some of the interleukin 6  [ **IL6** ]  gp130  [ **IL6ST** ]  molecular interactions into a two helix bundle structure was investigated.  Ref: 12630694 J Pept Sci, 2003
